# Heterochromatin: an epigenetic point of view in aging

**DOI:** 10.1038/s12276-020-00497-4

**Published:** 2020-09-04

**Authors:** Jong-Hyuk Lee, Edward W. Kim, Deborah L. Croteau, Vilhelm A. Bohr

**Affiliations:** 1 Laboratory of Molecular Gerontology, National Institute on Aging, National Institutes of Health, Baltimore, MD 21224 USA; 2 Danish Center for Healthy Aging, University of Copenhagen, 2200 Copenhagen, Denmark

**Keywords:** Chromosomes, Neurological disorders

## Abstract

Aging is an inevitable process of life. Defined by progressive physiological and functional loss of tissues and organs, aging increases the risk of mortality for the organism. The aging process is affected by various factors, including genetic and epigenetic ones. Here, we review the chromatin-specific epigenetic changes that occur during normal (chronological) aging and in premature aging diseases. Taking advantage of the reversible nature of epigenetic modifications, we will also discuss possible lifespan expansion strategies through epigenetic modulation, which was considered irreversible until recently.

## Aging: changes in genome infrastructure

Biochemical pathways that alter the physical organization of the genome could play a prominent role in age-related cellular degeneration. Chromosomal DNA is wound around proteins known as histones; when such assemblies are packed closely together, the genes in compacted regions are generally silenced. Jong-Hyuk Lee, Vilhelm A. Bohr and colleagues at the US National Institutes of Health, Baltimore, have reviewed evidence linking these ‘heterochromatin’ regions of the genome to the aging process. They find that diseases associated with premature age-related tissue degeneration are frequently marked by abnormal activity in genes involved in heterochromatin remodeling. Several biochemical and genetic factors that have been shown to experimentally reverse biological aging in experimental settings also appear to affect heterochromatin remodeling. Closer examination of the relevant remodeling processes could therefore reveal fundamental biological drivers of age-related degeneration.

## Introduction

Aging results from complex biological processes that are fundamental to all living organisms. Characterized by a gradual loss of molecular fidelity after reaching sexual maturity, aging leads to the functional loss of cells and tissues and ultimately causes the disease and death of an organism^1^. Aging significantly increases susceptibility to cancer, neurodegeneration, cardiovascular diseases, and metabolic disorders^2–4^. Although many studies have focused on the genetic factors that directly impact aging, nongenetic regulation of aging has recently gained interest as an important player in understanding the process of aging. Nongenomic changes that influence gene expression and alter the structural organization of chromatin are referred to as epigenetic alterations, which are broadly defined as alterations in modes of genomic regulation not directly encoded in DNA. Epigenetic alterations are profoundly involved in the process of aging, resulting in alterations and disturbances in the broad genome architecture and the epigenomic landscape^2^.

In this review, we discuss key epigenetic changes during chronological (normal) aging and premature aging diseases, focusing on the age-related loss of heterochromatin, and we discuss the current progress in the development of interventions for its amelioration or reversal.

## Histone and chromatin structure

DNA encodes essential information for maintaining organismal homeostasis. The total length of human genomic DNA in every cell is almost 2 m^5^, and a cell must pack this genomic DNA into its nucleus, which is only 6 μm in diameter. The packing ratio can reach ~10,000 to 1 when the naked DNA is packed into metaphase chromosomes^6^. Higher-order eukaryotes, including humans, have solved this problem through the use of histone proteins. Strands of DNA wrap around these core histones to form ‘beads on a string’ structures, referred to as nucleosomes. Each of these nucleosomes is composed of DNA wound 1.65 times around eight core histone (H2A, H2B, H3, and H4) proteins. Nucleosomes undergo folding and form a 30 nm chromatin fiber with loops of 300 nm in length. The 300 nm fibers are further compressed and folded to produce 250-nm-wide fibers that are tightly coiled into the chromatid of a chromosome^7^.

In addition to their function in packing genomic DNA into nuclei, histones also play a structural role in regulating gene expression. Histone tails protrude from their N-terminus into the nucleoplasm and are sites of diverse posttranslational modifications^8^. These modifications alter the overall electrostatic nature of the histones and affect the interactions with DNA, thereby regulating their binding^9^. When the binding affinity between histones and DNA is increased, chromatin is condensed into a closed conformation and prevents the access of transcription factors to the DNA, leading to gene repression. In contrast, a decreased binding affinity between histones and DNA leads to chromatin decondensation and an “open” conformation, granting transcription factors access to the DNA, resulting in transcription activation. In certain cases, histone modifications act indirectly and recruit effector proteins to activate downstream signaling pathways^10^, block the entry of chromatin remodeling complexes^11^, and influence the recruitment of other chromatin-modifying enzymes^12^ or transcription factors^13^.

In eukaryotes, higher-order chromatin can be categorized into two major structures. Heterochromatin is initially formed and regulated in embryogenesis and exhibits a condensed structure and an inactive transcription status. Euchromatin presents a decondensed chromatin structure and an active transcription status. Heterochromatin can be further subcategorized into constitutive and facultative heterochromatin. Constitutive heterochromatin refers to more permanent domains of heterochromatin that are predominantly located in centromeric or telomeric regions containing satellite sequences and transposable elements^14^. Satellite sequences are small regions that are repeated many times, and transposable elements are DNA sequences that can “jump” around the genome, play roles in cellular functions and are particularly prone to DNA double-strand breaks (DSBs) and nonallelic homologous recombination (NAHR) during meiosis^15^. DSBs and NAHRs can lead to chromosome rearrangements, a hallmark of cancers and hereditary diseases. The tightly packed constitutive heterochromatic state of these regions suppresses DSBs and NAHR events to protect the genome against harmful chromosomal rearrangements causing genomic instability^16^.

Facultative heterochromatin contains genes whose expression must be regulated according to specific morphogenesis or differentiation signals^17^. The regions of DNA packed into facultative heterochromatin can vary by cell type even within a species, so a DNA sequence that is packed into facultative heterochromatin in one cell could occur within euchromatin in another cell.

Cellular genomes are continuously damaged by reactive oxygen species through various processes^18^. An in vitro study showed that the binding of histones to DNA and their organization into higher-order chromatin structures dramatically protects DNA against hydroxyl radical-induced DNA strand breaks as well as some types of radiation-induced damage^19–21^. This suggests that heterochromatin contributes to cellular defense against the induction of oxidative DNA damage^22,23^. Cells in which heterochromatic proteins are knocked down or forced chromatin decondensation is induced are susceptible to increased DNA damage^24,25^. However, the high compaction of heterochromatic domains also makes them less accessible to DNA repair factors when DNA damage occurs^26^. Thus, heterochromatin must first become decondensed and take on euchromatic characteristics to allow transcription factors and/or DNA damage repair proteins access to the DNA^27^. Biochemical studies have shown that poly-ADP-ribosylating proteins (PARPs) are recruited to the sites of DNA damage and consume NAD^+^ to generate poly-ADP-ribosyl (PAR) chains, facilitating nucleosome disassembly by PARylating histones, which results in chromatin relaxation, and provides recruiting signals for DNA damage repair proteins^28^. Thus, it is clear that heterochromatin plays critical roles in development, transcription, DNA repair and the maintenance of genomic stability.

## The heterochromatin loss model of aging

The age-related dysregulation of chromatin organization can lead to cellular malfunctions and exacerbate the aging process^1,2,29^. One of the earliest models proposing the involvement of epigenetics in aging was the ‘heterochromatin loss model of aging^30,31^. This model suggests that heterochromatin domains established early in embryogenesis are broken down during aging, causing global nuclear architecture changes, the derepression of silenced genes, and aberrant gene expression patterns.

Increasing evidence supports this model. The loss of heterochromatin with aging has been documented across many model organisms, characterized by reduced heterochromatin markers or molecular markers associated with the formation and maintenance of heterochromatin. For example, the trimethylation of the ninth lysine on H3 histones (H3K9me3), which encourages DNA to bind more tightly to the histone complex and form heterochromatin, is gradually lost during the aging process.

Cellular models of aging also display enlarged nuclei, a sign of nuclear DNA decondensation and heterochromatin loss. Furthermore, experiments conducted across diverse organisms have provided evidence of disrupted transcriptional silencing due to heterochromatin loss during chronological aging^30–34^. The major changes in chromatin markers during organismal/cellular aging are listed in Table 1.

**Table 1:** Major chromatin marker changes during aging.

Component	Changes during aging	Molecular function	Organism	Reference
H2A	Decrease (cellular senescence)	Core histone	H.sapiens	^87^
H2B	Decrease (cellular senescence)	Core histone	H.sapiens	^87^
H3	Decrease (cellular senescence)	Core histone	H.sapiens	^87–89^
H4	Decrease (cellular senescence)	Core histone	H.sapiens	^87,88^
HP1 alpha	Decrease (organismal)	Heterochromatin component	H.sapiens	^38,42^
H3K9me3	Decrease (organismal)	Heterochromatin mark, transcription repression	H.sapiens	^90^
H3K27me3	Decrease (cellular senescence)	Repressive marker on bivalent promoter	H.sapiens	^89^
H4K20me3	Decrease (cellular senescence)	Pericentromeric heterochromatin	H.sapiens	^88^
H3K4me3	Remodeling (cellular senescence)	Active transcription on promoter	H.sapiens	^89^
H3K36me3	Remodeling (organismal)	Active transcription on gene body	D.melanogaster	^91^
H3K56ac	Decrease (cellular senescence)	DNA replication, nucleosome assembly	H.sapiens	^88,92^
H3K16ac	Decrease (organismal)	Telomere silencing	H.sapiens	^93^

Heterochromatin loss also occurs during senescence, a mechanism through which aging cells suppress their own ability to grow and divide in response to shortened telomeres, genomic instability, or the activation of oncogenes to evade malignancy^35,36^. Domains of facultative heterochromatin referred to as senescence-associated heterochromatin foci (SAHF) increase in senescent cells, silencing genes that promote cell division^37^. Although SAHFs increase during senescence, senescent cells still experience global heterochromatin loss, characterized by the loss of heterochromatic gene silencing and the subsequent expression of genes that were previously inaccessible to transcription factors^30^. Thus, despite the increase in and redistribution of localized facultative heterochromatin, the chromatin changes observed during senescence also seem to support the heterochromatin loss model of aging.

In addition to chronological aging, genome-wide heterochromatin loss has been reported in models of premature aging diseases. A summary of the characteristics of normal and premature aging diseases is provided in Table 2, and these characteristics are discussed in detail below.

**Table 2:** Features of normal and premature aging.

Diagnosis	Lifespan	Clinical features	Cause of death	Associated genetic mutation	Affected heterochromatin Component	Reference
Normal aging	78.7	• Vision/hearing loss • Cardiovascular disease/Hypertension • Osteoporosis • Osteoarthritis • Dementia • Diabetes mellitus • Immunosenescence • Cancer	Various	–	See Table 1	See Table 1^94^
HGPS	14.5	• Disproportionately large head • Narrow nasal ridge • Alopecia • Loss of subcutaneous fat • Progressive joint contractures • Low-frequency conductive hearing loss	• Atherosclerosis • Myocardial infarction • Heart failure • Cerebrovascular disease	LMNA	Decreased HP1 Decreased H3K9me3/H3K27me3	^36,95^
WS	54	• Loss and graying of hair • Hoarseness • Bilateral ocular cataracts • Type 2 diabetes mellitus • Hypogonadism • Skin ulcers • Osteoporosis • Cancer	• Myocardial infarction • Cancer	WRN	Decreased HP1 Decreased H3K9me3/H3K27me3	^42,96^
CS	16	• Cachectic dwarfism • Loss of subcutaneous fat • Progressive impairment of vision, hearing, central and peripheral nervous system • Congenital cataracts • Atherosclerosis	• Pneumonia/Respiratory ailments • Renal failure	• ERCC1 • ERCC4 • ERCC6 • ERCC8	Decreased H3 Decreased H3K9me3	^50,63,97,98^

**Fig. 1:**
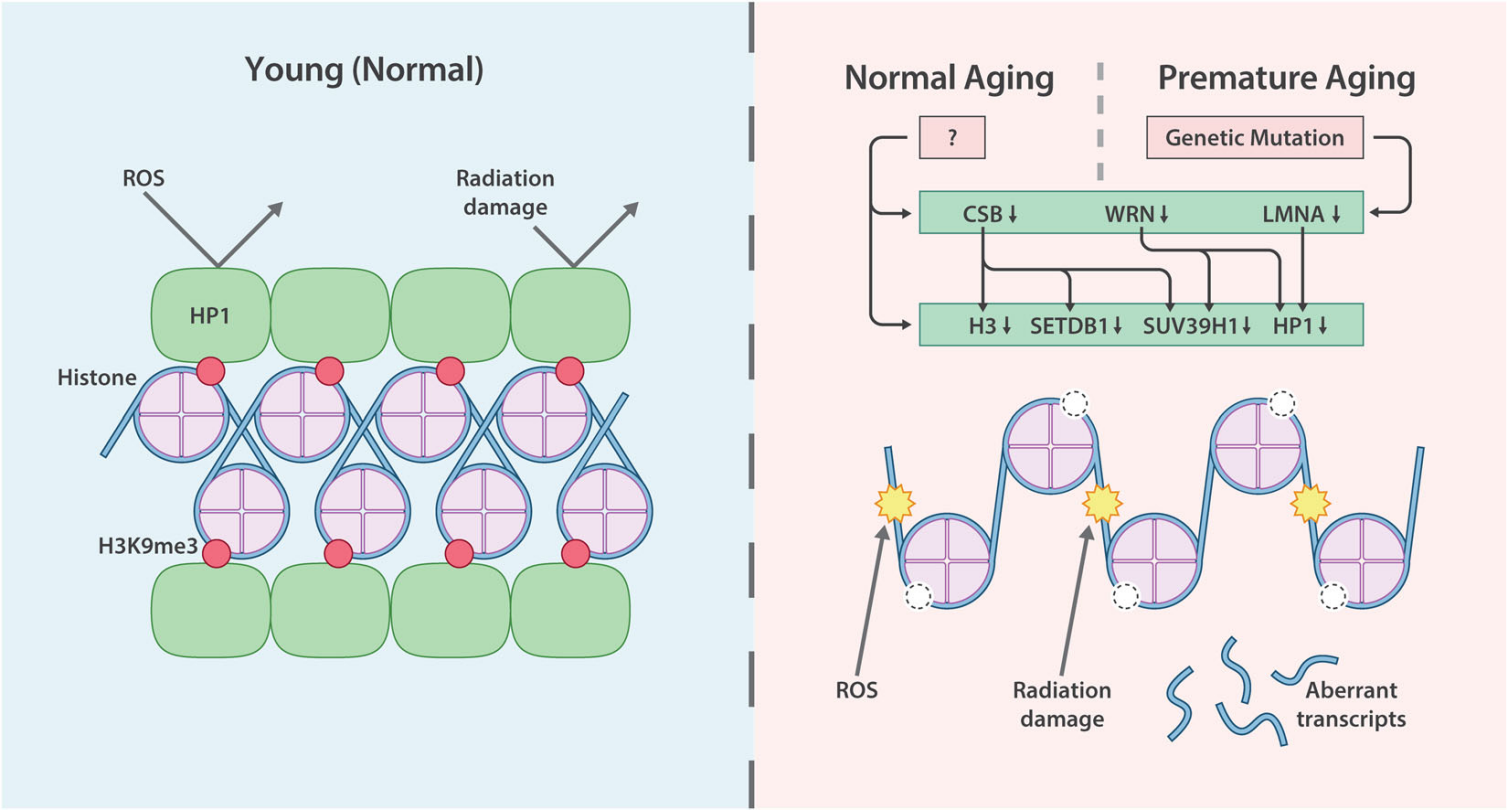
Overview of heterochromatin loss during aging. In young and healthy individuals, cells exhibit intact heterochromatin, a high level of H3K9me3, and a high level of HP1 bound to H3K9me3, which are factors that stabilize the heterochromatin complex. However, both chronologically and prematurely aged cells show decreased expression of core histones and reduced levels of H3K9me3 and HP1. CSB, WRN, and LMNA deficiency leads to H3, SETDB1, SUV39H1, and HP1 dysregulation, resulting in heterochromatin loss, DNA damage accumulation, and the expression of aberrant transcripts. The mechanisms under the decreased expression of CSB, WRN, and LMN with normal aging remain unclear.

Hutchinson–Gilford progeria (HGPS) is a rare autosomal-dominant genetic disorder caused by germline mutations in the *LMNA* gene, which encodes a component of the nuclear envelope. Affected patients have symptoms that resemble aspects of aging and typically live until only their mid-teens or early twenties. Cells cultured from these patients exhibit an abnormal nuclear morphology, including enlarged nuclei, an indication of heterochromatin loss^38^. Biochemical experiments also confirmed a decrease in H3K9me3 and reduced expression of HP1^39^, which is a protein that recognizes and binds to H3K9me3 to form a stable heterochromatin complex, in these cells^40^ (Fig. 1).

Werner syndrome (WS) is an accelerated aging disorder caused by mutations in the DNA repair gene *WRN*^41^. WRN-depleted mesenchymal cells from mice and chronologically aged human primary mesenchymal stem cells (MSCs) both show significantly decreased expression of the major H3K9me3 histone methyltransferase (HMTase) SUV39H1, a protein regulating the formation and maintenance of heterochromatin^42^. WRN knockout MSCs also show an abnormal nuclear envelope, enlarged nucleus, and decreases in pericentromeric and telomeric heterochromatin, which are characteristics observed in both chronological aging and HGPS patient cells. Catalytically inactive SUV39H1 knock-in in wild-type MSCs recapitulates the accelerated cellular senescence characteristics of WRN-deficient MSCs, suggesting that SUV39H1 plays an important role in the WRN-associated pathway for maintaining heterochromatin. Moreover, the overexpression of the H3K9me3-binding protein HP1α rescues heterochromatin disorganization and premature senescence in WRN-deficient MSCs.

The “Horvath Clock” describes a pattern of DNA methylation alteration that is currently a promising molecular marker for monitoring aging and predicting the life expectancy of humans^43^. The model also considers the global decline in genomic CpG methylation as the predominant event in aging, which is well documented in many studies^44–46^. As DNA and H3K9 methylation are strongly associated in mammals^47^, there is likely a causative link between these two phenomena^30,48,49^. However, the mechanisms underlying age-related DNA methylation changes and their relationship with heterochromatin loss during aging remain unclear.

## CSB: a new player in the heterochromatin loss model of aging

Cockayne syndrome (CS) is an autosomal recessive genetic disorder caused predominantly by genetic mutations in the *ERCC8* (CSA) or *ERCC6* (CSB) gene^50^; it is characterized by early-onset neurodegeneration, growth failure, impaired nervous system development, abnormal photosensitivity, vision defects, and premature aging. Both CSA and CSB genes are considered to be critical in transcription regulation^51^ and DNA repair^52^. CS patients also display characteristics of normal aging such as impaired mitophagy and accumulation of damaged mitochondria^53^.

Interestingly, the clinical features of CSA and CSB patients are similar, but the two proteins share no functional/structural homology. CSA is a WD-40 repeat-containing protein that functions as an adapter in an E3-ubiquitin ligase complex^50^. Biochemical experiments confirmed that CSA forms a complex with DDB1 (DNA damage-binding protein 1), Cul4 (cullin 4A), and Rbx1 (ring box 1), suggesting that CSA may be involved in the targeted ubiquitination of proteins^54,55^. CSB is a DNA-dependent adenosine triphosphatase SWI2/SNF2 family member implicated in chromatin remodeling during transcription^56^. CSB binds to DNA as a dimer and shortens the DNA contour length by actively wrapping or unwrapping DNA in the presence of ATP^57^. This ATP-dependent chromatin remodeling activity affects the DNA binding affinity of various proteins to their cognate binding sites on DNA. CSB facilitates the sequence-independent association of p53 with chromatin when p53 protein concentrations are low through its interaction with the C-terminal region of p53^58^. In vitro experiments confirmed that CSB can remove TFAM from double-stranded DNA and stimulate mitochondrial RNA polymerase elongation and transcription^59^. It has also been shown that CSB-deficient CS patient cells exhibit a defect in mitochondrial transcription, suggesting that CSB may be a factor involved in mtDNA nucleoid remodeling. Under conditions of oxidative stress, CSB colocalizes with the CCCTC-binding transcription factor in a subset of genomic regions to modulate chromatin structure and coordinate gene expression in response to oxidative stress^60^. Moreover, NAP1 (nucleosome assembly protein 1)-like histone chaperones facilitate the ATP-dependent chromatin remodeling activity of CSB for efficient transcription-coupled DNA repair^61^. Taken together, these studies suggest that CSB plays a central role in epigenetic signaling and that elucidating these pathways may be key to understanding characteristics of premature aging in CS pathology. Although epigenetic changes have been widely accepted as a marker of aging^62^ and a diagnostic marker in premature aging disorders, including HGPS^40^ and WS^42^, they have not been linked to CS until recently.

The immunofluorescence imaging and biochemical analysis of fibroblast cells from CS patients carrying a truncation mutation in CSB (CSB-deficient cells) showed enlarged nuclei, decreased H3K9me3 levels, and decreased expression of histone H3^63^. These findings are consistent with previous observations of abnormal nuclear morphology and decreases in heterochromatin markers in aged cells and cells from HGPS and WS patients^40,42^.

One of the key molecular characteristics of CSB-deficient cells is persistent PARP activation^64^, leading to the accumulation of PAR chains on damaged DNA and cellular NAD^+^ depletion, which is associated with aging and mitochondrial dysfunction^65^. The bioinformatic analysis of the PAR and H3K9me3 distribution in CSB-deficient cells revealed significantly increased PAR levels in transcription start site (TSS) regions and decreased H3K9me3 in TSS and constitutive heterochromatin regions^63^. Because TSS regions can be located in facultative heterochromatin, CSB-deficient cells likely experience decreases in facultative as well as constitutive heterochromatin^63^. The study suggested that the failed MDM2-mediated proteasomal degradation of activating transcription factor (ATF3), which usually binds to the promoter of the major facultative heterochromatin HMTase *SETDB1* to repress its transcription, may be the cause of heterochromatin loss in CSB-deficient cells. Indeed, forced expression of SETDB1 normalizes H3K9me3 levels and suppresses PAR accumulation in CSB-deficient cells.

Finally, on the basis of revealing significantly decreased CSB and SETDB1 protein expression levels with increasing age in fibroblasts from healthy human donors and mouse brains, the study concluded that CSB deficiency may contribute to heterochromatin loss during normal aging as well. In agreement, another study using normal human lung fibroblasts during replicative senescence detected the depletion of CSB^66^. The authors attributed this finding to the hypoacetylation of histone H3 at the CSB promoter. Thus, previous research shows that CSB plays a central role in heterochromatin loss in CS and even in normal aging through its chromatin remodeling activity and/or transcriptional regulation/interaction as well as interaction with epigenetic factors.

## Lifespan expansion strategies through epigenetic modulation

Interventions that take advantage of the malleability and reversibility of age-associated epigenetic alterations have been a topic of enthusiastic research. For example, in yeast, it has been shown that rescuing the age-related loss of histone proteins through overexpression dramatically extends lifespan^67^. It has also been shown in fruit flies that decreasing heterochromatin levels result in a shortening of lifespan, whereas the recovery of this reduction in heterochromatin levels by HP1 overexpression prolongs lifespan^33^.

Since the historic discovery of Yamanaka factors (OCT4, SOX2, KLF4, and MYC, also known as OSKM) by Takahashi et al.^68,69^, cellular reprogramming has been extensively studied, and many follow-up studies have reported that global epigenetic landscape reprogramming contributes to cell fate conversion and lineage determination. Simply overexpressing these four transcription factors in aged cells rejuvenates the cells to a younger state. Using this cellular reprogramming technique, fibroblasts and keratinocytes from individuals with various progeroid syndromes, such as WS, CS, xeroderma pigmentosum, ataxia telangiectasia, Fanconi anemia, dyskeratosis congenita, HGPS, Nestor–Guillermo progeria syndrome, and from healthy centenarians^70^ have been successfully dedifferentiated to iPSCs, though with a reduced reprogramming efficiency in some cases^71,72^. These studies suggest that the cellular state can be reset to a younger stage by epigenome reprogramming.

It has also been shown that partial reprogramming via the short-term cyclic expression of OSKM ameliorates cellular and physiological hallmarks of aging and prolongs lifespan in an HGPS mouse model^73^. The short-term induction of OSKM in these cells restored the decreased levels of H3K9me3 and downregulated histone γ-H2AX focus formation, a marker of nuclear DNA DSBs. Interestingly, H3K9 methyltransferase inhibitor treatment prevented the restoration of H3K9me3, and no amelioration of DNA damage was observed^73^. In CSB-deficient cells, the recovery of H3K9me3 by SETDB1 restoration suppressed PAR accumulation and rescued mitochondrial dysfunction^63^, a hallmark of chronological aging and other age-related diseases such as Alzheimer’s disease (AD)^74,75^. Taken together, the available results indicate that it is likely that H3K9me3 and the formation of heterochromatin are critical for DNA damage suppression.

Additional interventions are also effective in reversing the age-related changes in the epigenetic landscape. One of these interventions is dietary restriction. Accumulating evidence across multiple species suggests that caloric restriction is an effective dietary intervention for lifespan expansion and delaying signs of aging in many organisms. Evidence suggests that the biological effects of caloric restriction are closely interconnected with chromatin structure. A study in yeast showed that a histone deacetylase protein from the sirtuin family, Sir2P, plays a major role in regulating the effect of caloric restriction on chromatin structure^76^. In humans, the mammalian Sir2 ortholog, SIRT1, is activated by intracellular NAD^+^ and deacetylates histones, which is a prerequisite for heterochromatinization^77^. Moreover, SIRT1 upregulates SUV39H1 activity directly by deacetylating residue K266, present in the catalytic SET domain^78^. This suggests that sirtuins also play a role in protecting cells from heterochromatin loss by upregulating HMTases.

Since SIRT1 is activated by intracellular NAD^+^, NAD^+^ supplementation may also be an attractive intervention strategy for lifespan extension. Studies show that NAD^+^ supplementation reduces the symptoms of CS^64,79^, WS^80^ and AD^81^. It is believed that the reversal of intracellular NAD^+^ depletion contributes to recovery from impaired mitochondrial biogenesis, which gives rise to aging phenotypes in these cells^65^.

NAD^+^ is a central substrate of pathways crucial to healthy cellular function, such as the PARP1- and PARylation-dependent DNA damage-sensing and repair pathway, and the sirtuin family of histone deacetylases, which play important roles in maintaining heterochromatin, DNA repair, inflammation control, and antioxidative defense^82^. Thus, NAD^+^ becomes a valuable cellular commodity as it is increasingly depleted with the age-associated accumulation of DNA damage and mutations^83^. In addition, NAD^+^ supplementation through the provision of precursor molecules such as nicotinamide riboside or nicotinamide mononucleotide can be a powerful intervention for ameliorating age-associated deficits in key cellular processes^84^.

Interestingly, the levels of almost all sirtuins increase as an effect of caloric restriction, and it is thought that sirtuins mediate at least some of the beneficial effects of this dietary intervention^82^. Caloric restriction has been shown to delay the age-related loss of facultative heterochromatin in flies, a process in which sirtuins play an important role. Importantly, experiments in which flies were rapidly switched between an unrestricted diet and dietary restriction showed that they undergo rapid changes in gene silencing levels, demonstrating the epigenetic plasticity of chromatin during aging and the speed at which such alterations can occur and, thus, the translational potential of epigenetically focused interventions^85^.

The effect of dietary restriction on chromatin structure has also been replicated in human normal fetal lung fibroblasts, in which glucose restriction induced increases in H3K4me2 and H3K9me3 at *hTERT* (human telomerase reverse transcriptase) and the *p16INK4a* promoter, respectively. Through this glucose restriction-induced chromatin remodeling, the cellular lifespan was extended via *hTERT* expression and *p16INK4a* suppression^86^.

While these studies show that caloric restriction and NAD^+^ supplementation exhibit important connections to the regulation of both longevity and chromatin structure, it is currently unclear whether the observed chromatin changes are direct consequences associated with lifespan extension. To answer this question, a direct molecular connection between organismal aging and caloric restriction-mediated chromatin remodeling needs to be established.

## Conclusion

The crucial roles of heterochromatin in protecting the genome from DNA damage and regulating gene expression are clear. Significant supporting evidence has been obtained for the heterochromatin loss model of aging, first proposed in 1997, which has further associated age-related heterochromatin loss with various other hallmarks of aging, such as mitochondrial dysfunction and cellular senescence. Although the loss of gene silencing is proposed as one such mechanism, much work remains to be done to elucidate the precise mechanisms underlying the influence of heterochromatin loss not only on chronological aging but also on age-related diseases such as cancers, cardiovascular diseases, and neurodegenerative diseases such as AD.

Due to their reversibility and malleability, the targeting of epigenetic alterations associated with aging shows remarkable promise, either through lifestyle-oriented (caloric restriction) or pharmaceutical interventions. Understanding the mechanistic basis of already proven interventions such as caloric restriction will help us to better understand age-related heterochromatin loss and how to prevent it.

Models of premature aging diseases such as CS and WS provide excellent approaches for teasing out the specific pathways involved in more fundamental mechanisms of age-related epigenetic alterations. Previous studies in such models have identified key components of these mechanisms and their functions in either exacerbating or mediating age-related heterochromatin loss, which has led to a more detailed understanding of chronological aging. Research that utilizes these models to understand the role of heterochromatin and its implications for other hallmarks of aging will continue to be vital to our understanding of aging itself.
